# Comparative Evaluation of Digital Panoramic and Digital Intraoral Periapical Radiographic Techniques for the Assessment of Interproximal Bone Loss: A Cross-Sectional Study

**DOI:** 10.7759/cureus.98061

**Published:** 2025-11-29

**Authors:** Sabahat Hafiz Khan, Saad O Alazmi, Salman T Ansari, Sharjeel Khan

**Affiliations:** 1 Department of Dentistry, Maharashtra Public Health Services, Nagpur, IND; 2 Department of Periodontology and Implant Dentistry, College of Dentistry, Qassim University, Buraydah, SAU; 3 Department of Forensic Medicine and Toxicology, N.K.P. Salve Institute of Medical Sciences and Research Centre, Nagpur, IND

**Keywords:** digital panoramic radiography, interproximal bone loss, intraoral periapical radiography, intrasurgical measurements, periodontitis

## Abstract

Introduction

Accurate assessment of interproximal bone loss is essential for diagnosing and managing chronic periodontitis, a leading cause of tooth loss. Digital panoramic and intraoral periapical radiography are widely used, but their diagnostic accuracy, compared to intrasurgical measurements, remains debated. This study evaluated the reliability of these digital radiographic techniques in assessing interproximal bone loss in patients with moderate to advanced chronic periodontitis.

Methodology

An observational cross-sectional study was conducted with 35 patients (23 females and 12 males, aged 30-50 years) with moderate to advanced chronic periodontitis. Digital panoramic radiographs (ORTHOPHOS XG; Sirona, Bensheim, Germany) and intraoral periapical radiographs (SuniRay; Suni Medical Imaging, San Jose, CA, USA, long-cone paralleling technique) were obtained for 117 posterior teeth (234 sites). Bone loss was measured from the cemento-enamel junction to the alveolar crest and compared with intrasurgical measurements (gold standard) using a UNC-15 periodontal probe (Hu-Friedy, Chicago, IL, USA). Intraclass correlation coefficients (ICC), sensitivity, specificity, and chi-square tests were used to assess agreement and diagnostic accuracy.

Results

Both radiographic techniques showed almost perfect agreement with intrasurgical measurements (ICC 0.97 for panoramic and 0.98 for intraoral). Mean bone loss was 5.9957 ± 2.08 mm (panoramic), 5.8932 ± 2.10 mm (intraoral), and 5.7607 ± 2.07 mm (intrasurgical), with no significant differences (p > 0.05). Both methods slightly overestimated bone loss (0.235 mm for panoramic and 0.132 mm for intraoral). Sensitivity was 77.8% (panoramic) and 76.7% (intraoral), with specificities of 98.7% and 97.4%, respectively. Bilateral symmetry was significant (ICC 0.8 for the maxilla and 0.73 for the mandible).

Conclusion

Digital panoramic and intraoral periapical radiography are highly accurate for assessing interproximal bone loss, with digital panoramic radiography offering a viable alternative to intraoral radiography due to comparable accuracy, reduced radiation, and enhanced patient comfort. Future research should explore anterior quadrants and three-dimensional imaging.

## Introduction

Periodontal disease, a chronic inflammatory condition affecting the supporting structures of teeth, is a leading cause of tooth loss worldwide [1]. Chronic periodontitis, characterized by the progressive loss of alveolar bone and periodontal attachment, is primarily caused by plaque-induced microbial infections and is exacerbated by factors such as smoking, diabetes, and poor oral hygiene [2]. The accurate assessment of alveolar bone loss is critical for diagnosis, treatment planning, and monitoring disease progression, as bone loss is a hallmark of periodontal destruction [3].

Radiographic imaging is a cornerstone of periodontal diagnosis, providing non-invasive visualization of bone levels and defect morphology [4]. Intraoral periapical radiographs, using techniques such as the long-cone paralleling method, offer high resolution and are considered the standard for assessing interproximal bone loss, due to their ability to capture detailed anatomical structures [5,6]. However, they are limited by their small field of view, requiring multiple exposures, which increases radiation dose and patient discomfort [7]. Digital panoramic radiography, in contrast, provides a comprehensive view of the maxillofacial region in a single image, reducing radiation exposure and acquisition time [8]. Despite its advantages, panoramic radiography is often criticized for lower resolution, geometric distortion, and superimposition of structures, which may compromise its accuracy in detecting subtle bone changes [9].

The advent of digital radiography has enhanced both intraoral and panoramic imaging by improving image quality, enabling post-processing, and reducing radiation doses compared to conventional film-based methods [7]. Digital intraoral sensors, such as charge-coupled devices (CCDs), offer high spatial resolution (up to 22 lp/mm), while panoramic systems, like the ORTHOPHOS XG, provide reliable imaging with standardized protocols [7,10]. However, the diagnostic accuracy of these digital modalities for periodontal bone loss remains debated, particularly when compared to intra-surgical measurements, which serve as the gold standard for assessing true bone levels [11]. Previous studies have reported varying sensitivity and specificity for radiographic detection of bone loss, with intraoral radiographs generally outperforming panoramic images, though results are inconsistent due to differences in methodology and defect severity [12].

Despite advances in radiographic technology, there is a paucity of studies directly comparing digital panoramic and intraoral radiography with intrasurgical measurements in patients with moderate to advanced chronic periodontitis. Most research has focused on either radiographic modality in isolation or has used conventional radiography, which may not reflect the capabilities of modern digital systems [6,13,14]. Furthermore, the reliability of these methods in detecting osseous defect severity and bilateral symmetry, a recognized feature of periodontal disease [15], remains underexplored. Hence, this study was conducted to address these gaps by evaluating the diagnostic accuracy and reliability of digital panoramic and intraoral radiography in assessing interproximal bone loss, using intrasurgical measurements as the reference standard, to guide clinical decision-making in periodontal therapy. We hypothesized that both techniques would show almost perfect agreement (primary outcome: intraclass correlation coefficient (ICC) ≥ 0.95; mean bone loss difference <1 mm, as the clinical equivalence margin) with intrasurgical measurements at the interproximal site level (234 sites from 117 posterior teeth), with secondary outcomes including sensitivity, specificity, and bilateral symmetry.

## Materials and methods

After approval from the Institutional Ethical Committee (reference number SDKSDCH/PG/40/2022) and after obtaining written informed consent from the patients, an observational cross-sectional study was performed at the Outpatient Department of Oral Medicine and Radiology for a duration of 18 months. The inclusion criteria for patients consisted of: (1) patients with moderate to advanced chronic periodontitis, (2) radiographic evidence of bone loss, and (3) patients with posterior teeth present bilaterally. The exclusion criteria involved pregnant females, patients with intraoral appliances interfering with clinical or radiographic evaluation, and patients with bilaterally missing posterior teeth. Based on the eligibility criteria, a total of 35 patients (23 females and 12 males), aged 30-50 years, suffering from moderate to advanced chronic periodontitis and requiring periodontal flap surgery, were included. The diagnosis of chronic periodontitis was based on clinical and radiographic evidence of bone loss, as defined by the 2017 World Workshop on the Classification of Periodontal and Peri-Implant Diseases and Conditions [16].

The materials used for clinical examination consisted of a disposable mouth mask and gloves, a sterile mouth mirror, a probe, an explorer, tweezers, a kidney tray, cotton pellets, gauze pieces, and an UNC-15 periodontal probe (Hu-Friedy, Chicago, IL, USA). For radiographic evaluation, a lead apron and thyroid shield, a dental intraoral X-ray machine, the SuniRay Dental Digital X-ray imaging system (Suni Medical Imaging, San Jose, CA, USA) with SuniRay imaging software [7], an intraoral digital sensor (size 2, CCD) [17], disposable plastic sleeves, the ORTHOPHOS XG X-ray system version 2.53 (Sirona, Bensheim, Germany) with “Sidexis” imaging software (Sirona, Bensheim, Germany), and a personal computer with monitors (resolutions: 1366 × 768 pixels for panoramic and 1024 × 768 pixels for intraoral) were utilized.

All patients underwent initial periodontal treatment (scaling) following a complete clinical examination, as recommended for managing chronic periodontitis. Intraoral examinations were conducted, and data were recorded using Russell’s Periodontal Index, a validated tool for assessing periodontal disease severity based on clinical signs, such as gingivitis and pocket formation [18]. Oral hygiene instructions were provided to all patients to standardize baseline conditions. Radiographic examinations included digital panoramic radiographs and digital intraoral periapical radiographs of the posterior quadrants of the maxillary and mandibular arches on which surgery was planned. Prior to exposure to radiation, suitable patient protectors, such as a lead apron and thyroid shield, were used. 

Digital panoramic radiographs were obtained using the ORTHOPHOS XG X-ray system with a CCD-based line sensor (active sensor area: 138 × 6.48 mm, resolution: 0.027 mm pixel size, magnification: ~1:1.19) [7]. Exposure parameters were adjusted based on patient build (thin build: 62 kV, 8 mA, 14.1 sec; heavy build: 68 kV, 15 mA, 14.1 sec; extra heavy build: 73 kV, 15 mA, 14.1 sec) to optimize image quality and minimize radiation exposure. Digital intraoral periapical radiographs were captured using a size 2 CCD-based sensor (SuniRay; dimensions: 43.5 × 31.5 mm, active surface: 36 × 25.97 mm, resolution: 22 lp/mm, pixel size: 22 × 22 µm) [17], with a dental X-ray unit operating at 65 kVp and 8 mA, as per manufacturer recommendations. Posterior periapical radiographs were taken using the long-cone paralleling technique with Sensibles Universal sensor holders to ensure accurate projection geometry, with an exposure time of 0.08 sec. Disposable plastic sleeves covered the sensor to maintain infection control.

Radiographic measurements were taken from the cemento-enamel junction (CEJ) to the interproximal alveolar crest (AC) on both mesial and distal sites of each tooth in the posterior quadrants, consistent with established methods for assessing alveolar bone loss [14]. For digital panoramic radiographs, measurements were performed using the linear measurement tool in Sidexis software, following adjustments to brightness and contrast as needed to enhance visualization of landmarks (e.g., CEJ and AC), per standard protocols [13]. For digital intraoral periapical radiographs, measurements were conducted using SuniRay software, with similar brightness and contrast adjustments applied. Supplementary anterior quadrant measurements were also recorded using digital intraoral periapical radiographs to assess symmetry in bone loss, as symmetry has been noted in periodontal disease progression [15]; however, these were not included in the primary analysis, which focused on posterior sites. Exclusion criteria for measurements included third molars, overhanging restorations, missing teeth, unrecognizable CEJ, proximal caries, and root stumps, as these factors can obscure accurate assessment [14]. If both mesial and distal surfaces were visible and included in the surgical procedure, the tooth was evaluated. The more apical AC level was scored when two levels were detected [13]. Measurements were recorded to the nearest 0.01 mm and rounded to 1 mm if ≥0.50 mm. A total of 117 teeth (234 sites) were evaluated. Radiographs were reassessed by the same observer under identical viewing conditions, as recommended for ensuring measurement reliability [19].

Periodontal flap surgeries were performed by skilled periodontal surgeons in the Department of Periodontology, following standard protocols, with patient consent obtained. After reflecting a full-thickness flap and removing granulation tissue, the distance from the CEJ to the interproximal AC was measured to the nearest 1 mm using a UNC-15 periodontal probe, before any osseous surgery or regenerative procedures, as this provides the gold standard for bone level assessment [20]. Osseous defects were classified based on the distance from the CEJ to the AC (mild: 1-3 mm, moderate: 4-6 mm, and severe: ≥7 mm), following established criteria for periodontal disease severity [13,16].

Data were analyzed using the Statistical Package for the Social Sciences (SPSS) version 16.0 for Windows 7 (Released 2007; SPSS Inc., Chicago, IL, USA). Descriptive statistics summarized age, sex, and the distribution of osseous defect sites by tooth group and jaw location. Normality of data was tested using the Shapiro-Wilk test, and the data were found to be normal. The chi-square test compared the severity of osseous defects detected intrasurgically and radiographically, as used in prior studies to evaluate categorical agreement. Paired t-tests compared mean interproximal bone loss measurements between surgical and radiographic methods in the maxillary and mandibular jaws, consistent with methods for assessing measurement differences. The ICC assessed the reliability of digital panoramic and intraoral radiography compared to intrasurgical measurements, with ICC values of 0.8-1 indicating almost perfect agreement [19,21]. Sensitivity and specificity were calculated for both radiographic techniques to evaluate diagnostic accuracy. Symmetry of bone loss between the left and right sides was evaluated using ICC for digital panoramic radiographs, as bilateral symmetry is a recognized feature of periodontal disease.

## Results

The study included 35 patients with moderate to advanced chronic periodontitis, comprising 12 males (34.3%) and 23 females (65.7%), with a male-to-female ratio of 12:23. The age range was 30-50 years, with a mean age of 38.17 ± 4.82 years. The mean age for males was 39.91 ± 4.83 years, and for females, 37.26 ± 4.66 years. A total of 117 teeth were evaluated and classified according to tooth group and jaw location for osseous defects, with 53 (45.29%) in the maxillary arch and 64 (54.70%) in the mandibular arch. These were categorized into premolars (55 teeth: 26 maxillary and 29 mandibular) and molars (62 teeth: 27 maxillary and 35 mandibular). Each tooth was assessed at mesial and distal sites, resulting in 234 sites (106 maxillary, 45.29%, and 128 mandibular, 54.70%). The site distribution included 110 premolar sites (52 maxillary and 58 mandibular) and 124 molar sites (54 maxillary and 70 mandibular). The distribution of osseous defects, depending on severity, as detected intra-surgically and on imaged radiography, is illustrated in Table 1.

**Table 1:** Distribution of osseous defects depending on severity as detected intra surgically and on imaged radiography 'S' indicates highly significant.

Radiography technique	Mild (1-3 mm)	Moderate (4-6 mm)	Severe (≥7 mm)	Total	Chi-square value	p-value
Digital panoramic	10 (4.28%)	157 (67.09%)	67 (28.63%)	234 (100%)	106.1	0.0001 (S)
Digital intraoral	10 (4.28%)	162 (69.23%)	62 (26.49%)	234 (100%)	114.5	0.0001 (S)
Intra-surgical	9 (3.85%)	169 (72.22%)	56 (23.93%)	234 (100%)	109.9	0.0001 (S)

The contingency analysis evaluated agreement in defect severity between methods, as demonstrated in Table 2. The chi-square test reported highly statistically significant results.

**Table 2:** Contingency analysis of digital panoramic radiographs by intra-surgical categorical assignments of alveolar crest level (no. of sites) 'S' indicates highly significant.

Digital panoramic radiography	Intra-surgical measurements				Chi-square value	p-value
	Mild	Moderate	Severe	Total	326.3	p < 0.001 (S)
Mild	7 (70.0%)	3 (30.0%)	0	10		
Moderate	2 (1.23%)	160 (98.76%)	0	162		
Severe	0	6 (9.67%)	56 (90.32%)	62		
Total	9	169	56	234		

Similarly, for AC levels, contingency analysis of the digital intraoral method, by intra-surgical categorical analysis, is reported in Table 3. The chi-square test reported highly statistically significant results.

**Table 3:** Contingency analysis of digital intraoral radiographs by intra-surgical categorical assignments of alveolar crest level (no. of sites) 'S' indicates highly significant.

Digital intraoral radiography	Intra-surgical measurements				Chi-square value	p-value
	Mild	Moderate	Severe	Total	327.2	p = 0.0001 (S)
Mild	8 (80.0%)	2 (20.0%)	0	10		
Moderate	2 (1.23%)	152 (93.82%)	8 (4.9%)	162		
Severe	0	3 (4.83%)	59 (95.16%)	62		
Total	10	157	67	234		

Moreover, for AC, contingency analysis of digital panoramic, by digital intraoral categorical analysis, is reported in Table 4. The chi-square test reported highly statistically significant results.

**Table 4:** Contingency analysis of digital panoramic radiographs by digital intraoral radiographs categorical assignments of alveolar crest level (no. of sites) 'S' indicates highly significant.

Digital panoramic radiography	Digital intraoral radiography				Chi-square value	p-value
	Mild	Moderate	Severe	Total	341.2	p < 0.001 (S)
Mild	8 (80%)	2 (20%)	0	10		
Moderate	2 (1.27%)	152 (96.81%)	3 (1.91%)	157		
Severe	0	8 (11.94%)	59 (88.05%)	67		
Total	10	162	62	234		

The mean interproximal bone loss was 5.9957 ± 2.08 mm for digital panoramic radiography, 5.8932 ± 2.10 mm for digital intraoral radiography, and 5.7607 ± 2.07 mm for intra-surgical measurements and is illustrated in Table 5. No statistically significant differences were observed between the three methods (one-way ANOVA, p > 0.05).

**Table 5:** Comparison of mean amount of interproximal bone loss between digital panoramic radiography, digital intraoral radiography, and intra-surgical measurements 'NS' indicates non-significant.

Radiography technique	Number of patients (N)	Mean (mm)	Standard deviation (mm)	One-way ANOVA (F-value)	p-value
Digital panoramic	234	5.9957	2.08	1.24	p = 0.51 (NS)
Digital intraoral	234	5.8932	2.10		
Intra-surgical	234	5.7607	2.07		
Total	702	5.8832	2.08		

The mean difference from intra-surgical measurements (gold standard) was 0.235 mm for the digital panoramic technique and 0.132 mm for the digital intraoral technique, as demonstrated in Table 6; however, both values were not statistically significant (unpaired t-test, p > 0.05).

**Table 6:** Comparison of mean difference of interproximal bone loss on digital panoramic and digital intraoral radiographic measurements with intra-surgical measurements 'NS' indicates non-significant.

Radiography technique	Mean (mm)	Mean difference (mm)	Unpaired t-test value	p-value
Digital panoramic	5.9957 ± 2.08	0.23504	t = 1.69	p = 0.315 (NS)
Digital intraoral	5.8932 ± 2.10	0.13248	t = 1.71	p = 0.329 (NS)

The mean dimension of AC levels of maxillary teeth, as assessed by intra-surgical measurement, was 5.52 ± 1.80 mm; by digital panoramic radiography, it was 5.69 ± 1.85 mm, and by digital intraoral radiography, it was 5.57 ± 1.89 mm. There was no statistically significant difference between the intra-surgical and digital panoramic, intra-surgical and digital intraoral, or digital panoramic and digital intraoral measurements. The mean dimension of AC of mandibular teeth, as assessed by intra-surgical measurement, was 5.95 ± 2.26 mm; by digital panoramic radiography, it was 6.24 ± 2.23 mm, and by digital intraoral radiography, it was 6.15 ± 2.22 mm. There was no statistically significant difference between the intra-surgical and digital panoramic, intra-surgical and digital intraoral, or digital panoramic and digital intraoral measurements, as illustrated in Table 7.

**Table 7:** Mean dimensions of alveolar crest levels as assessed by intra-surgical and radiographic evaluation according to their location 'NS' indicates non-significant.

Location	Intra-surgical (IS) (mm)	Digital panoramic (DP) (mm)	Digital intraoral (DI) (mm)	IS vs DP		IS vs DI		DP vs DI	
				Unpaired t-test value	p-value	Unpaired t-test value	p-value	Unpaired t-test value	p-value
Maxillary	5.52 ± 1.80	5.69 ± 1.85	5.57 ± 1.89	0.56	p > 0.05 (NS)	0.81	p > 0.05 (NS)	0.73	p > 0.05 (NS)
Mandibular	5.95 ± 2.26	6.24 ± 2.23	6.15 ± 2.22	0.41	p > 0.05 (NS)	0.52	p > 0.05 (NS)	0.71	p > 0.05 (NS)

The digital panoramic radiography underestimated the bone loss at 11 sites when compared to the true value of the intrasurgical measurement. The mean value of this underestimation was 1.06 ± 1.44 mm, whereas it overestimated the bone loss at 57 sites, with the mean value of overestimation being 1.08 ± 0.03 mm. The digital intraoral radiographs underestimated the bone loss at 13 sites when compared to the true value of the intrasurgical measurement. The mean value of this underestimation was 0.16 ± 0.42 mm. There was an overestimation of bone loss at 40 sites, with the mean value of overestimation being 0.99 ± 0.03 mm, as illustrated in Table 8.

**Table 8:** Comparison of digital panoramic and digital intraoral radiography with intrasurgical measurements of bone loss for underestimation and overestimation 'NS' indicates non-significant.

Radiographic methods	No. of sites of underestimation	Underestimation compared to true value (mm)	No. of sites of overestimation	Overestimation compared to true value (mm)	Unpaired t-test value	p-value
Digital panoramic	11	1.06 ± 1.44	57	1.08 ± 0.03	0.21	p = 0.67 (NS)
Digital intraoral	13	0.16 ± 0.42	40	0.99 ± 0.03	1.29	p = 0.31 (NS)

When the agreement between different methods for the estimation of interproximal bone loss was compared, it was found that, between digital panoramic and intra-surgical measurements, there was agreement of scores for 227 (97.5%) sites and disagreement at seven (2.5%) sites. Similarly, when digital intraoral and intra-surgical measurements were compared, there was agreement of scores for 230 (98.4%) sites and disagreement at four (1.6%) sites, as illustrated in Table 9.

**Table 9:** Measurement of agreement and disagreement between radiographic and intra-surgical measurements at alveolar crest levels

Methods compared	Agreement	Disagreement	Chi-square value	p-value
Digital panoramic - Intra-surgical	227 (97.5%)	7 (2.5%)	0.83	p = 0.36
Digital intraoral - Intra-surgical	230 (98.4%)	4 (1.6%)		

The reliability of the digital panoramic technique and the digital intraoral technique was evaluated by comparing them with the intra-surgical technique. The ICC for digital panoramic and intra-surgical was 0.97, which was statistically highly significant. The ICC for digital intraoral and intra-surgical was 0.98, which was also statistically highly significant. The ICC for digital panoramic and digital intraoral was 0.98, which was again statistically highly significant, as reported in Table 10.

**Table 10:** Reliability of DP radiography and DI radiography in estimating interproximal bone loss 'S' indicates highly significant. DP, Digital panoramic radiography; DI, Digital intraoral radiography; IS, Intra-surgical measurements; ICC, Intraclass correlation coefficient; CI, Confidence interval

Groups	ICC	95% CI	p-value
DP versus IS	0.97	0.96 - 0.98	< 0.001 (S)
DI versus IS	0.98	0.97 - 0.98	< 0.001 (S)
DP versus DI	0.98	0.97 - 0.98	< 0.001 (S)

The sensitivity of the digital panoramic technique in the assessment of interproximal bone loss was 77.8%, whereas that of the digital intraoral technique was 76.7%. The specificity of the digital panoramic technique was 98.7%, and that of the digital intraoral technique was 97.4%, as demonstrated in Table 11.

**Table 11:** Sensitivity and specificity of digital panoramic radiography and digital intraoral radiography for assessment of interproximal bone loss

Radiography technique	Sensitivity	Specificity
Digital panoramic	77.8%	98.7%
Digital intraoral	76.7%	97.4%

The agreement in orthopantomogram (OPG) readings from one side with the OPG readings from the contralateral side for assessing interproximal bone loss was calculated. When the interproximal bone loss on the maxillary right side was compared with that on the maxillary left side, there was agreement at 80% of sites, whereas 20% of sites were not in agreement. The ICC for the maxillary right and left sides was 0.8, suggestive of almost perfect agreement. Similarly, in the mandibular arch, the interproximal bone loss on the mandibular right side was compared with that on the mandibular left side; there was agreement at 73.9% of sites, whereas 26.1% of sites were not in agreement. The ICC for the mandibular right and left sides was 0.73, suggestive of moderate agreement, as demonstrated in Table 12.

**Table 12:** Reliability of digital panoramic radiography in estimating symmetry of interproximal bone loss ICC, Intraclass correlation coefficients

Interproximal bone loss	Agreement	Disagreement	ICC
Maxillary	80%	20%	0.80
Mandibular	73.9%	26.1%	0.73

## Discussion

This study evaluated the diagnostic accuracy of digital panoramic and intraoral periapical radiography in assessing interproximal bone loss in patients with moderate to advanced chronic periodontitis, using intrasurgical measurements as the gold standard. The results demonstrated that both radiographic techniques exhibited high agreement with intrasurgical assessments, with an ICC of 0.97 for digital panoramic and 0.98 for digital intraoral radiography, indicating almost perfect reliability [21-23]. The mean differences from intrasurgical measurements were minimal (0.235 mm for panoramic and 0.132 mm for intraoral), and neither was statistically significant, suggesting that both methods are clinically reliable for periodontal bone loss assessment.

Both radiographic techniques slightly overestimated bone loss compared to intrasurgical measurements, with digital intraoral radiography showing a smaller mean difference (0.132 mm) than digital panoramic (0.235 mm). This overestimation aligns with previous studies, such as Wolf et al. and Kim et al., which reported overestimations of 0.74-1.91 mm and 1.63-2.0 mm, respectively, for digital intraoral radiography [24,25]. However, the findings contrast with studies like Christiaens et al., Tonetti et al., and Eickholz et al., which noted underestimation of bone loss by digitized radiographs [26-28]. The overestimation observed in this study may be attributed to the high resolution of modern digital systems (e.g., 22 lp/mm for intraoral CCD sensors) and standardized imaging protocols, which enhance the visualization of bone margins but may amplify subtle defects [7,17]. For digital panoramic radiography, the overestimation is consistent with Pepelassi and Diamanti-Kipioti, who reported exaggerated defect depths in conventional panoramic images [14], but contrasts with studies like Kaimenyi and Ashley, and Akesson et al., which found underestimation, particularly in severe periodontitis [19,29]. Pepelassi and Diamanti-Kipioti suggested that the degree of bone loss influences radiographic accuracy, with underestimation in initial disease and overestimation in severe cases, which may explain the variability across studies [14].

The high sensitivity (77.8% for panoramic and 76.7% for intraoral) and specificity (98.7% for panoramic and 97.4% for intraoral) of both techniques underscore their diagnostic utility. Notably, digital panoramic radiography showed slightly higher sensitivity, aligning with Ivanauskaite et al., who reported greater sensitivity for panoramic compared to bitewing radiography [30]. However, this contradicts Pepelassi and Diamanti-Kipioti, who found intraoral periapical radiographs more sensitive [14]. The high specificity of digital panoramic radiography in this study contrasts with that of Molander et al., who reported lower specificity (35%) for conventional panoramic images [31]. The improved performance of digital panoramic systems, such as the ORTHOPHOS XG used here, may be due to advanced image enhancement tools (e.g., brightness/contrast adjustments) in Sidexis software for clearer landmark visualization, and minimized geometric distortion via CCD-based sensors (pixel size 0.027 mm; magnification ~1:1.19), which outperform analog film's susceptibility to superimposition and uneven exposure [7,8].

A significant finding was the almost perfect agreement between digital panoramic and intraoral radiography (ICC 0.98), particularly for moderate and severe osseous defects. This supports Persson et al. and Langlois et al., who found comparable accuracy between conventional panoramic and periapical radiographs [23,32]. The agreement was highest for moderate defects, suggesting that both techniques are most reliable in this range, possibly due to clearer delineation of bone margins compared to mild defects, where subtle changes are harder to detect, or severe defects, where complex defect morphology may complicate measurements [14]. The ability of digital panoramic radiography to achieve comparable accuracy to intraoral radiography, combined with its advantages, such as lower radiation dose, single-image acquisition, and greater patient comfort, supports its potential to replace intraoral radiography for assessing interproximal bone loss in posterior quadrants [33]. This is further corroborated by Haghgoo et al., who advocate panoramic radiography as a viable alternative to full-mouth intraoral surveys [34]. However, panoramic systems report limitations, including geometric distortion, magnification variability, and anatomical superimpositions, which can influence quantitative bone-level measurements by introducing minor distortions in complex morphologies [9,31]. In this study, these were minimized via protocol standardization and post-processing, yielding negligible impact on ICC and mean differences, yet they highlight the need for clinician awareness in interpreting subtle changes, particularly outside posterior regions.

The study also confirmed significant bilateral symmetry in interproximal bone loss, with almost perfect agreement in the maxillary arch (ICC 0.8) and moderate agreement in the mandibular arch (ICC 0.73). This aligns with Rise and Tollefsen, who demonstrated symmetrical patterns in periodontal disease progression, likely reflecting systemic host responses rather than localized factors alone [15]. The slightly lower agreement in the mandibular arch may be due to anatomical variations or differential plaque accumulation, as suggested by Mombelli and Meier [35]. This symmetry supports the use of digital panoramic radiography for comprehensive assessments, as it captures both arches in a single image, facilitating the evaluation of bilateral patterns.

Strengths, limitations, and future recommendations

The strengths of this study include its robust methodology, with standardized radiographic protocols, a well-defined patient cohort (35 patients and 234 sites), and intrasurgical measurements as the gold standard. The use of digital systems (ORTHOPHOS XG for panoramic and SuniRay for intraoral) ensured high image quality, and the direct measurement method enhanced reproducibility compared to indirect methods. However, limitations include the focus on posterior quadrants, limiting generalizability to anterior regions, where panoramic radiography's diagnostic reliability is further compromised by superimposition of the cervical spine, hindering accurate interpretation of bone levels [9], and the two-dimensional nature of the imaging modalities, which cannot fully capture three-dimensional defect morphology. Future research should explore anterior quadrants and incorporate advanced imaging techniques, such as cone-beam computed tomography (CBCT), which offers superior accuracy for complex defects. Additionally, larger sample sizes and longitudinal designs could further validate the findings and assess the impact of radiographic assessments on treatment outcomes.

## Conclusions

This study demonstrated that digital panoramic and intraoral periapical radiography are highly accurate for assessing interproximal bone loss in patients with moderate to advanced chronic periodontitis, showing almost perfect agreement with intrasurgical measurements. Both techniques slightly overestimated bone loss, but these differences were not statistically significant, affirming their clinical reliability. Digital panoramic radiography exhibited comparable diagnostic performance to intraoral radiography, alongside advantages of reduced radiation exposure and enhanced patient comfort, though its utility is tempered by inherent magnification and geometric distortion, which may affect quantitative precision in complex cases. The significant bilateral symmetry in bone loss further supports the use of panoramic radiography for comprehensive periodontal assessments. These findings suggest that digital panoramic radiography can serve as a practical complementary or preliminary alternative to digital intraoral periapical radiography for evaluating interproximal bone loss in posterior quadrants, offering a practical alternative for routine periodontal diagnostics, with intraoral imaging recommended for confirmatory, high-precision assessments. Future research should investigate the efficacy of these techniques in anterior quadrants, explore three-dimensional imaging modalities like CBCT, and employ larger cohorts to enhance generalizability and optimize diagnostic strategies for periodontal care.
